# A Comparison of Electronic Structure Methods for Predicting the Hydrogenation Energies of Candidate Molecules for Hydrogen Storage

**DOI:** 10.1021/acs.jpca.5c05284

**Published:** 2025-11-14

**Authors:** Amanda Dumi, Shiv Upadhyay, Hassan Harb, Rajeev S. Assary, Dan C. Sorescu, Kenneth D. Jordan, Anouar Benali

**Affiliations:** † Department of Chemistry, 6614 University of Pittsburgh, Pittsburgh, Pennsylvania 15260, United States; ‡ Material Science Division, 1291 Argonne National Laboratory, Argonne, Illinois 60439, United States; § United States Department of Energy, National Energy Technology Laboratory, Pittsburgh, Pennsylvania 15236, United States; ∥ Department of Chemical and Petroleum Engineering, 6614 University of Pittsburgh, Pittsburgh, Pennsylvania 15261, United States; ⊥ Computational Science Division, 1291 Argonne National Laboratory, Argonne, Illinois 60439, United States

## Abstract

The development of novel energy materials and fuels is required to expand current available energy sources. Aiming to reach this goal, there is growing interest in using molecular hydrogen as an energy carrier due to its abundance and high energy density. Liquid organic hydrogen carriers (LOHCs) are a promising route to the large-scale storage and transport of hydrogen for use in the energy economy. The search for thermodynamically viable LOHC molecules for real world use has led to a set of constraints on the dehydrogenation enthalpy and the minimum gravimetric hydrogen capacity. These constraints allow one to formulate the search for an ideal LOHC candidate molecule as an optimization problem well suited to the strengths of machine learning and artificial intelligence computational approaches. A critical barrier to a large-scale, high-throughput screening of LOHC candidate molecules is the lack of reliable training data. Computational electronic structure methods including density functional theory, coupled cluster approximations, and diffusion Monte Carlo can be used to provide training data where experimental data are either unreliable or do not exist. In this work, we use these methods to calculate the dehydrogenation energies and enthalpies of candidate LOHC molecules.

## Graphical abstract

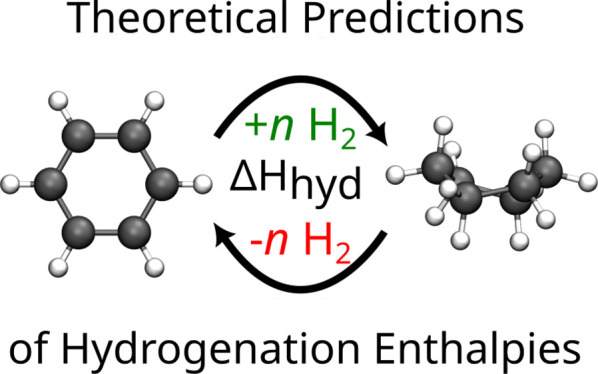

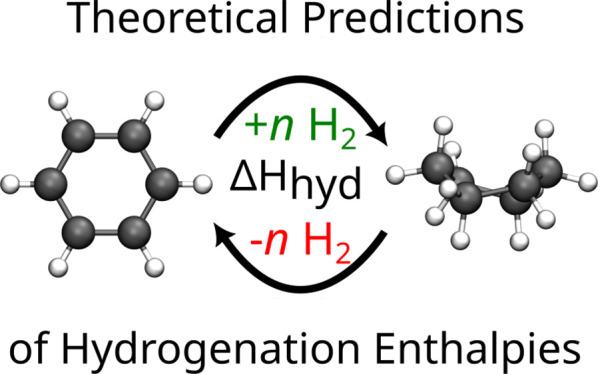

## Introduction

The development of novel energy materials and fuels is required to meet the need to move toward affordable and reliable energy sources.^1^ To drive affordable and resilient energy fuels, interest in molecular hydrogen as an energy carrier has been growing due to its abundance and high energy density.^2,3^ The concept of using molecular hydrogen as an energy carrier has been around for decades, and the term “hydrogen economy” was coined in 1972 by Bockris and Appleby to refer to the network of producing, transporting, and using hydrogen on a large scale.^4,5^ However, large-scale storage and transport of molecular hydrogen remains challenging. An alternative approach of chemically storing hydrogen in unsaturated liquid hydrocarbon molecules called LOHCs has gained recent interest.^2,6–8^ LOHCs are unsaturated organic compounds that can chemically bind and release molecular hydrogen in a catalyst-mediated cycle of hydrogenation and dehydrogenation, under specific reaction conditions.^2,6,9,10^ By being implemented in a recyclable manner, LOHCs can contribute to a resilient and affordable energy system.^10–12^ LOHCs, with their high hydrogen storage capacity and energy density, are suitable for portable applications and offer a better safety profile than hydrogen gas, making them ideal for transportation and safety-critical uses.^9,13–18^

The selection of an ideal LOHC requires careful consideration of several critical properties.^2,19^ Primary among these is the dehydrogenation enthalpy, which should ideally fall within the range of 9.6–16.7 kcal/mol H_2_.^20,21^ This range, based on multiple experimental studies of previously reported LOHCs, ensures that the hydrogen-rich LOHC can retain hydrogen over extended periods, yet not so tightly that it cannot be readily released when needed.^19^ Another major consideration is the requirement of a minimum gravimetric hydrogen capacity of 5.5% wt H_2_, a condition set forth by the U.S. Department of Energy (DOE) to ensure a basic level of energy efficiency in potential LOHCs.^22^ Beyond these energy-related parameters, the kinetics of the LOHCs must also be efficient, with the ability to undergo multiple hydrogenation and dehydrogenation cycles facilitated by a suitable catalyst.^6,10,23–25^ Finally, the selection process also accounts for several other key factors including the material’s commercial availability, toxicity, stability, flammability, and its ability to exist in a liquid state at room temperature.^26–30^ All these considerations, while individually important, collectively dictate the suitability and performance of a molecule as a LOHC.

Prominent LOHC dehydrogenated/hydrogenated pairs, such as benzene/cyclohexane,^31,32^ toluene/methyl cyclohexane,^31^ and *N*-ethylcarbazole/perhydro-*N*-ethylcarbazole^33,34^ have been extensively investigated for large-scale hydrogen storage and transportation due to their favorable attributes such as low dehydrogenation enthalpies (within the desired range of 9.6–16.7 kcal/mol H_2_) and high hydrogen storage capacities, exceeding the DOE’s 5.5% threshold.^2,6,15^ However, these systems are accompanied by several limitations: for instance, benzene is carcinogenic *N*-ethylcarbazole is a solid at room temperature with a melting point of 68 °C, posing practical application challenges,^2,35^ and toluene and methyl cyclohexane, are classified as highly flammable due to their low flash points of −3 and 6 °C, respectively.^26^ Further compounding these limitations are the catalytic cycles for hydrogenation and dehydrogenation, which often occur under high temperature and pressure conditions mediated by precious metal catalysts. This limits their effectiveness, safety, and practicality.^10^ Additionally, several LOHC systems are prone to side reactions and degradation processes and experience less than complete hydrogenation, which results in a hydrogen storage capacity below theoretical expectations, thereby diminishing their reusability and overall efficiency.^2^ These challenges highlight the need for the discovery of new, efficient, and sustainable LOHCs.

The computational exploration of the vast chemical space of unsaturated organic molecules presents a formidable challenge, making it a prime candidate for machine learning (ML) techniques to facilitate the rapid evaluation of numerous potential molecules.^36–38^ Many studies rely on density functional theory (DFT) to generate training data ML models. While DFT methods have been used to characterize LOHCs in previous studies^39,40^ and for reaction energies in general,^41^ there is no guarantee DFT will be adequate for unexplored forms of LOHCs. In fact, in previous studies it was shown that commonly employed DFT approaches can exhibit errors of several kcal/mol in hydrogenation enthalpies, which hinders the identification and appraisal of viable LOHC molecules.^41–43^ To enhance the discovery of LOHCs, accurate quantum chemical methods that consistently predict hydrogenation energies correctly to within 1 kcal/mol per molecule of H_2_ are imperative.^44^

In cases where DFT falls short, many-body methods such as diffusion Monte Carlo (DMC)^45–47^ and low-scaling coupled cluster methods emerge as powerful tools that can achieve a balance of accuracy and computational feasibility. The applicability of DMC to ML efforts has been demonstrated by Tenti et al.^48^ and Huang et al.^49^ Additionally, DMC has been successfully used to describe hydrogen reactions on surfaces.^50–52^ Low-scaling coupled cluster methods have been used in assessing conformer energies,^53^ and creating reference databases for organic molecules.^54^

In this work, we explore the performance of ab initio methods including DMC and domain based local pair-natural orbital coupled cluster singles, doubles, and perturbative triples (DLPNO-CCSD­(T))^55^ to predict the hydrogenation energies and enthalpies of a series of molecules that are of interest as LOHCs but for which the ωB97X-D,^56^ B3LYP,^57–60^ and MO6–2X^61^ DFT functionals have proven inadequate.^42^ Since the performance of these density functional approximations varies, we also calculated the hydrogenation energies and enthalpies of this set of molecules with the ωB97M-V functional.^62^ This functional was selected because of its exhaustive parametrization process^62^ and its recent successes on similar bond-breaking processes.^63–65^

## Theoretical Methods

### Molecule Selection and Generation

To construct a data set suitable for benchmarking, we sourced molecules that were utilized as a starting set for LOHC discovery in a previous study.^42^ The primary rationale behind the selection of these molecules was their known experimental dehydrogenation enthalpies, an essential parameter for understanding their efficacy as LOHCs. Additionally, the majority of the molecules in this set have been previously identified as potential candidates for LOHC systems.^42^ In cultivating the data set, the Simplified Molecular Input Line Entry System (SMILES) strings^66,67^ reported in Table S1 of the Supporting Information were converted to 3D structures using the RDKIT package^68^ and were optimized using the universal force field (UFF) method.^69^ For systems where multiple conformers were possible further refinement was done by first optimizing the geometries of the various conformers using the Frequency, Geometry Noncovalent, eXtended tightbinding (GFNN-xTB)^70^ DFT method together with the conformer–rotamer ensemble sampling tool (CREST) driver.^71^ In cases that the UFF and CREST calculations gave two or more low-energy conformers, these structures were reoptimized at the ωB97X-D/6-31G­(2df,p)^72–74^ level of theory, and the lowest energy conformer from these calculations was used for the subsequent calculations unless noted otherwise. For a subset of the molecules DLPNO-CCSD­(T) calculations were also carried out using the ωB97M-V geometries, with results summarized in the Supporting Information. These calculations gave hydrogenation energies very close to those obtained using the B3LYP optimized geometries.

We recognize potential uncertainties in reported experimental dehydrogenation values due to various factors. Contemporary calorimeters are subject to errors arising from calibration uncertainties, thermal leakages, or baseline drifts; deviations during calibration, for instance, that can distort enthalpy measurements.^75–78^ Challenges also persist in fully saturating LOHCs with hydrogen, as highlighted by studies showing dibenzyl toluene’s hydrogen loading reaching only about 90% of its theoretical capacity. Furthermore, LOHCs are susceptible to side reactions, especially at high hydrogenation/dehydrogenation temperatures. Molecules such as *N*-ethyl carbazole can undergo ancillary reactions due to suboptimal conditions, catalyst inefficiencies, or the tendency of *N*-alkyl substituents in carbazoles to dissociate prematurely. This, along with the presence of multiple conformers and unaccounted low-lying isomers with varying thermodynamics, can skew the actual enthalpy measurements. Finally, variability in experimental parameters such as temperature and pressure can further introduce discrepancies in the reported data.

### Density Functional Theory Calculations

Starting from the geometries in the previous section, the geometries of all species considered were reoptimized all-electron using both the B3LYP^57–60^ and ωB97M-V^62^ density functional methods, together with the cc-pVQZ basis set^79,80^ obtained from the Basis Set Exchange.^81–83^ These calculations were performed using the PySCF software version 2.1.0.^84,84–86^ The coupled cluster and DMC calculations used the B3LYP-optimized geometries. For a subset of the molecules domain based lower pair-natural orbital (DLPNO)-coupled cluster singles, doubles, and perturbative triples (CCSD­(T)) calculations were also carried out using the ωB97M-V geometries, with results summarized in the Supporting Information. These calculations gave hydrogenation energies very close to those obtained using the B3LYP optimized geometries.

In addition, harmonic vibrational frequencies were calculated at the ωB97M-V/aug-cc-pVTZ^87,88^ level of theory using geometries optimized with this basis set and functional. These calculations were performed using the ORCA version 5.0 software.^89^ The harmonic frequencies were used to obtain the estimates of the vibrational zero-point energies (ZPEs) and thermal corrections to facilitate comparison with experiment. The rigid rotor approximation was also used in obtaining the thermal corrections. A summary of these corrections can be found in Table S5. Unscaled vibrational frequencies were used for ZPE and thermal corrections. The scaling of the computed vibrational frequencies to approximately account for anharmonic effects was found to have a minimal impact on the computed hydrogenation energies, and this analysis is included in the Supporting Information.

### Coupled-Cluster Methods

For all species considered, hydrogenation energies were calculated using the DLPNO-CCSD­(T) method, treating all electrons and employing the frozen-core approximation and the cc-pVQZ basis set. CCSD­(T) calculations with the local natural orbital (LNO) and DLPNO approximations were found to give similar energetics.^90,91^ A summary of this comparison can be found in Table S2. In addition, the energetics of the C_6_H_6_ + 3H_2_ → C_6_H_12_ reaction was calculated using the LNO-coupled cluster singles, doubles, and triples (CCSDT)^90^ method, in which the triples term is treated nonperturbatively. Due to the computational demands of the LNO-CCSD­(T) and LNO-CCSDT calculations they were carried out with the smaller cc-pVDZ basis.^79,80^ Finally, a PNO-space extrapolation was conducted to assess the impact of the PNO-space truncation.^92,93^ The extrapolation had little impact on the hydrogenation energy for most systems (∼0.01 kcal/mol mean change over the test set) and was unstable for four of the systems studied here. Therefore, we chose to use DLPNO calculations at a single (TightPNO) threshold. All coupled cluster calculations were performed using ORCA version 5.0 ^89^ with the LNO-CCSDT calculations utilizing the MRCC software interface.^94,95^

### Quantum Monte Carlo Methods

DMC^45^ is a real-space stochastic approach to solving the many-body Schrödinger equation. The method is particularly attractive given its low scaling with the number of electrons and the highly parallelizable algorithms. Being a real space method, DMC energies are much less sensitive to the choice of the atomic basis set than methods working in a space of Slater determinants.

When dealing with fermionic particles, the DMC method requires the use of the fixed-node approximation^96^ to maintain the antisymmetric property of the wave function. For efficient sampling and to reduce statistical fluctuations, Slater-Jastrow trial wave functions are used.^97^ The nodes of the trial wave function are set through one or more Slater determinants comprised of single-particle orbitals, which in this work are expanded in a B-spline basis. Single determinant (SD) trial wave functions for DMC calculations made use of B3LYP orbitals, while the multideterminant (MD) trial wave functions were generated by the configuration interaction using a perturbative selection (CIPSI)^98^ procedure with iteratively refined natural orbitals.

To reduce the cost of the DMC calculations as well as to reduce the fluctuations near the ionic core regions, the core-correlated electron core potential (ccECP) pseudopotentials were used for all elements.^99^ The pseudopotentials replaced the 1*s* electrons for period two elements (e.g., C, O, N), but no electrons were replaced for H. These pseudopotentials were designed to be used with high-accuracy many-body methods such as DMC. To address errors introduced by the use of nonlocal pseudopotentials a modified version of the T-moves algorithm, denoted as ‘v3′ in the QMCPACK user’s manual,^100^ was used.

The SD DMC calculations were carried out using trial wave functions expanded in both the ccECP-cc-pVTZ and ccECP-cc-pVQZ basis sets. The dehydrogenation energies obtained from these two sets of calculations are nearly identical, so for the SD-DMC calculations only the results with the ccECP-cc-pVQZ basis set are reported here. Also, considering this close agreement, only the smaller ccECP-cc-pVTZ basis set was used for the multideterminant DMC calculations.

As noted above, the trial wave functions for the DMC calculations also include Jastrow factors that are symmetric with electron exchange and reduce the variance. The Jastrow factors contain terms for one-body (electron–ion), two-body (electron–electron) and three-body (electron–electron–ion) interactions. In this study, 10 parameters were employed per spin-channel for the one- and two-body Jastrow factors and 26 parameters for the three-body Jastrow factor. Since many molecules were considered in this work, the Jastrow cutoff distances were not optimized for each system. Instead default distances were fixed to 10 Bohr for the one- and two-body terms and 5 Bohr for the three-body term for all systems. The parameters in the Jastrow function were optimized by minimizing the energy using the linear method^101^ in variational Monte Carlo (VMC). The DMC calculations were done using a time step of 0.001 Ha^–1^. The quantum Monte Carlo (QMC) calculations were done in QMCPACK version 3.17.^102,103^

### Selected Configuration Interaction Calculations

To reduce the fixed-node errors in DMC energies, one can employ trial wave functions comprised of multi-Slater determinants rather than just one. Here we use CIPSI, which is a selected configuration interaction (SCI) method, to identify a compact determinant expansion, while not requiring the full Hilbert space. In this work, the quality of the nodal surface is explored by comparing DMC energies for a single Slater determinant trial wave function to those employing multi-Slater determinant expansions of various sizes as generated by CIPSI. The initial CIPSI calculations were performed with a single Slater determinant wave function of Hartree–Fock (HF) orbitals and employing ccECP pseudopotentials and the corresponding ccECP-cc-pVTZ basis set. The multideterminant expansions were created by first generating small expansions and forming natural orbitals to use in generating larger expansions. Various trial wave functions are constructed by truncating the large expansion according to the magnitude of the configuration interaction (CI) coefficients. For generating the truncated trial wave functions the following values were used to screen the magnitude of the CI coefficient: 1 × 10^–2^, 1 × 10^–3^, 1 × 10^–4^. The CIPSI calculations were done using Quantum Package 2.^104^

### Definition of the Hydrogenation Energy

The hydrogenation energies per H_2_ molecule are calculated as

$$\Delta E_{\mathrm{hyd}}=\frac{E_{h}-(E_{\mathrm{dh}}+N_{H_{2}}\times E_{H_{2}})}{N_{H_{2}}}$$ 1

where *E*_h_ (*E*_dh_) is the total energy of the hydrogenated (dehydrogenated) species, *N*_H_2__ denotes the moles of H_2_, and *E*_H_2__ is the energy of a single H_2_ molecule. These energies were corrected for vibrational ZPE and thermal corrections calculated at *T* = 298.15 K and compared with experimental values of the enthalpies of dehydrogenation (Table S1). To enhance the discovery of LOHCs, accurate quantum chemical methods that consistently predict hydrogenation energies correctly to within 1 kcal/mol per molecule of H_2_ are imperative.

## Results and Discussion

In the following sections, we compare the performance of DLPNO-CCSD­(T) against less approximate coupled cluster methods. We then use DLPNO-CCSD­(T) as the reference for the hydrogenation energies to explore the performance of the B3LYP and ωB97M-V DFT methods and of SD-DMC. For selected systems, we examine the impact of orbital optimization of the SD trial wave functions on the DMC energies. We examine improvements in the DMC hydrogenation energies upon adoption of MD trial wave functions. Finally, the ZPE and thermally corrected results are compared with experiment.

### Performance of DLPNO-CC Methods for Hydrogenation Energy Prediction

For the subset of molecules for which we carried out both LNO-CCSD­(T) and DLPNO-CCSD­(T) calculations, we find that the two methods give hydrogenation energies that agree to within 0.3 kcal/mol per H_2_ molecule (see Figure S1 and Table S2 in the Supporting Information). In addition, for the benzene + 3H_2_ → cyclohexane reaction we find that the DLPNO-CCSD­(T)/cc-pVDZ hydrogenation energy is close to that from the LNO-CCSDT/cc-pVDZ calculations in which the triples are treated nonperturbatively (agreeing to within 0.2 kcal/mol per H_2_). We also established that the DLPNO-CCSD­(T) values of the hydrogenation energies calculated with the cc-pVQZ basis set are close to those obtained with the cc-pVTZ basis set, leading us to conclude that the DLPNO-CCSD­(T) hydrogenation energies are well converged with the cc-pVQZ basis set. When corrected for vibrational ZPE and thermal effects, the computed value of the benzene hydrogenation energy per H_2_ molecule is within 0.5 kcal/mol of the experimental value, which will be further discussed in a following section. These results lead us to conclude that the DLPNO-CCSD­(T)/cc-pVQZ calculations are appropriate for providing benchmark numbers for assessing the performance of the other theoretical approaches considered.

### Assessing the Performance of DMC and DFT Methods for Δ*E*_hyd_

In this section, the performance of SD-DMC, B3LYP, and ωB97M-V DFT methods to predict hydrogenation energies is explored. Figure 1 reports the differences between the hydrogenation energies from the DFT and SD-DMC calculations and the results of the DLPNO–CCSD­(T) calculations. The performance of each method in terms of their Mean Absolute Error (MAE)­s for the entire data set are summarized in Table 1. The dehydrogenation energies from the SD-DMC calculations have a MAE of 1.7 kcal/mol per H_2_ molecule compared to the DLPNO–CCSD­(T) results. For comparison, we note that the corresponding MAEs for the B3LYP and ωB97M-V functionals are 2.53 and 0.26 kcal/mol, respectively.

**Figure 1:**
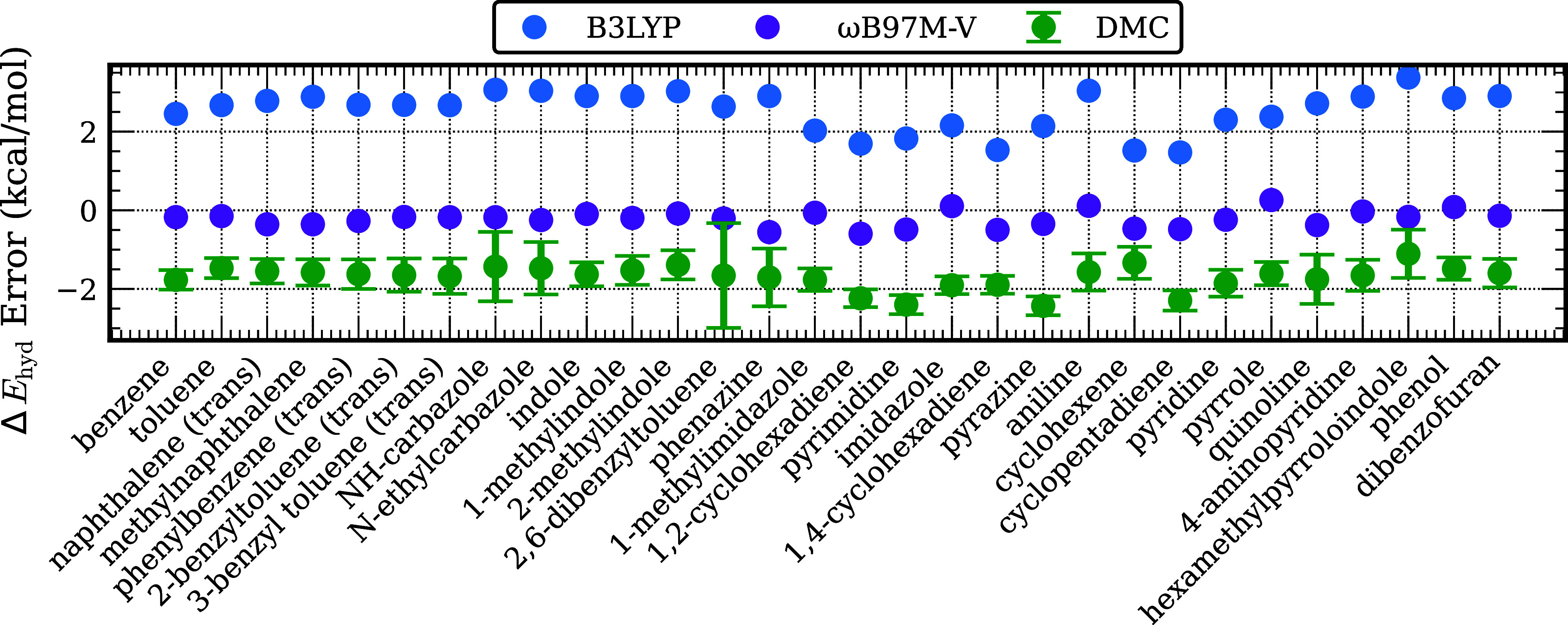
Signed error with respect to the DLPNO-CCSD­(T) reference of Δ*E*_hyd_ values from SD-DMC (green circles) and the B3LYP (black circles) and ωB97M-V (purple circles) calculations. The “(trans)” label indicates that the trans isomer of the hydrogenated species was used.

**Table 1:** Summary of the MAE in the Δ*E*_hyd_ Values from ωB97M-V, B3LYP, and SD-DMC as Compared with DLPNO-CCSD­(T) for the 30 Systems in the Test Set^a^

method	MAE kcal/mol
ωB97M-V	0.26
B3LYP	2.53
SD-DMC	1.70

a These results are without ZPE or thermal corrections.

#### Multideterminant Trial Wave Functions for DMC

As noted above, the SD-DMC hydrogenation energies differ on average by about 1.70 kcal/mol per H_2_ from the DLPNO-CCSD­(T) values. Moreover, in all cases, the SD-DMC calculations give more negative hydrogenation energies than the DLPNO-CCSD­(T) calculations. The major source of this discrepancy is expected to be fixed node errors in the SD-DMC calculations which are expected to be higher in the unsaturated than in the saturated compounds.

Figure 2 reports for benzene and cyclohexane the CIPSI variational energy with the second-order perturbative correction, *E*_var._ + *E*_PT2_, as a function of the number of Slater determinants in the variational space as well as the DMC energy as a function of the number of Slater determinants in the trial wave function. As expected, the CIPSI *E*_var._ + *E*_PT2_ energies converge slowly with increasing number of determinants. However, as the number of determinants is increased from one to about 1000, the CIPSI *E*_var._ + *E*_PT2_ energy drops more rapidly for benzene than for cyclohexane. While the DMC energies show more rapid convergence, they are still not well converged when employing 2 × 10^6^ Slater determinants in the trial wave functions. Moreover, while the DMC energy of cyclohexane is nearly constant as the trial wave function grows from 1 to ∼6 × 10^4^ determinants and then undergoes an appreciable energy lowering upon further expansion to ∼10^6^ determinants, the DMC energy of benzene steadily decreases as the number of Slater determinants is increased, with an appreciable energy lowering in going from 1 to 500 determinants in the trial wave function. These results illustrate the challenge in balancing the nodal surface error when comparing two different chemical species.

**Figure 2:**
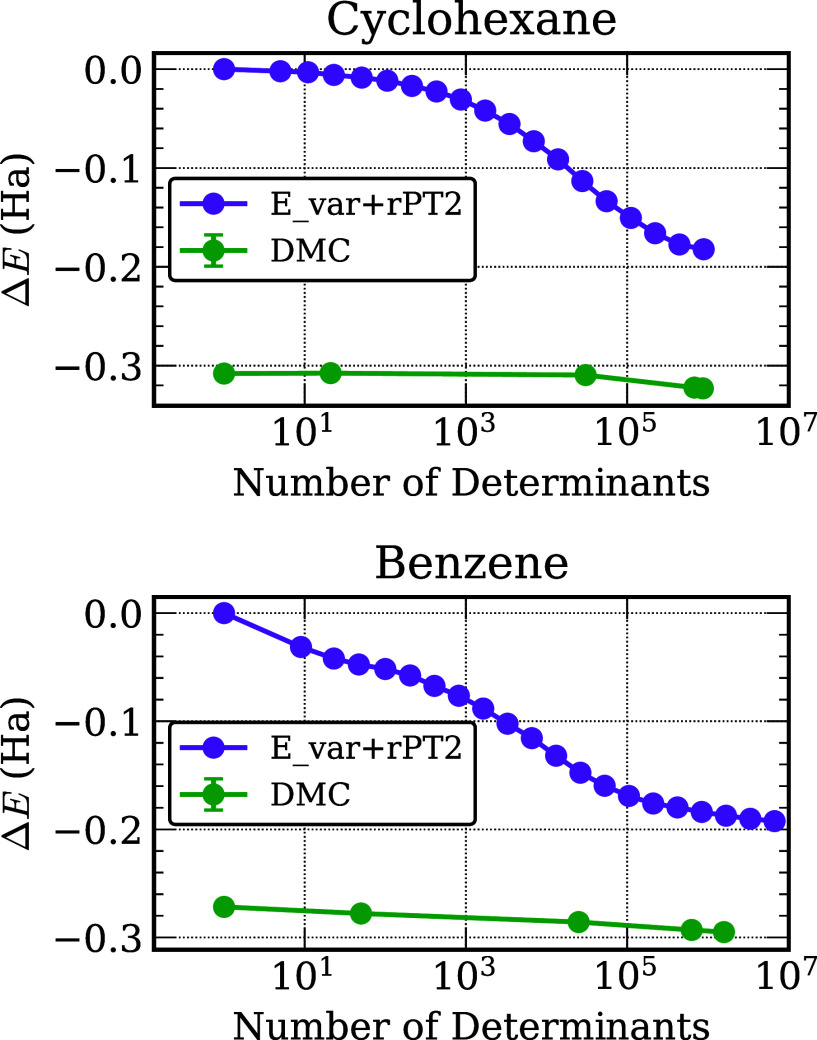
Change in the CIPSI and DMC energies of benzene and cyclohexane as a function of the number of Slater determinants. The energies are reported relative to the single determinant HF energy.

One approach to balance wave function quality for two systems is variance matching.^105^ Figure 3 reports the variance per electron for the CIPSI variational energies of benzene and cyclohexane as a function of the number of Slater determinants generated using the CIPSI method. As seen in the figure, the variance per electron of benzene with a threshold giving ∼100 SDs for the trial wave function is approximately equal to that of cyclohexane with only one Slater determinant.

**Figure 3:**
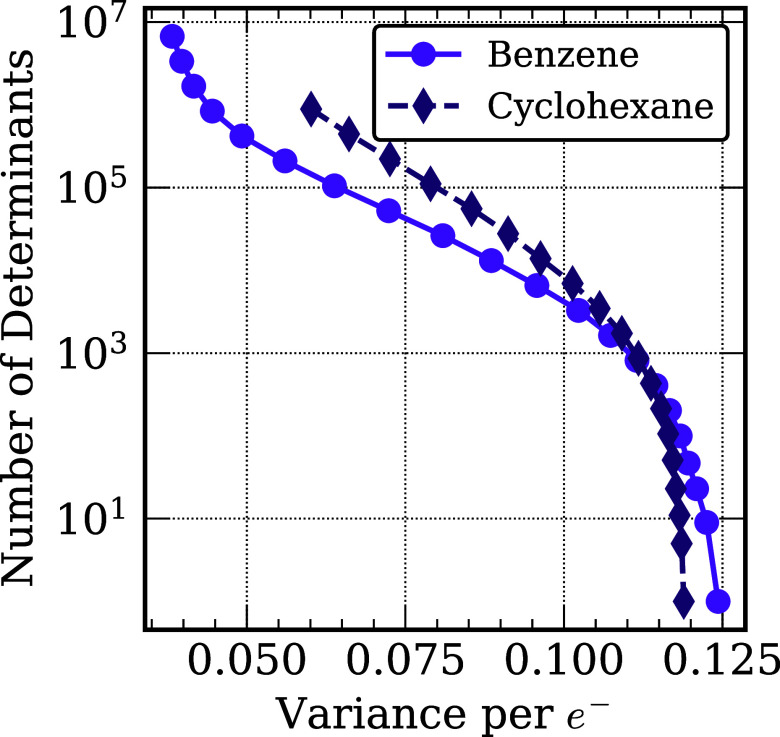
Variance per electron of the CIPSI energies as a function of the number of Slater determinants for benzene and cyclohexane.

Additionally, we explored the variance per electron in the DMC energies of benzene and cyclohexane as a number of Slater determinants in the trial wave functions with the results being reported in Figure 4. We note that the variance of H_2_ is negligible and is not included. It is seen from this figure that for benzene one needs to employ ∼1000 Slater determinants in the MD-DMC calculations to achieve the same variance per electron as obtained for the SD-DMC calculations on cyclohexane.

**Figure 4:**
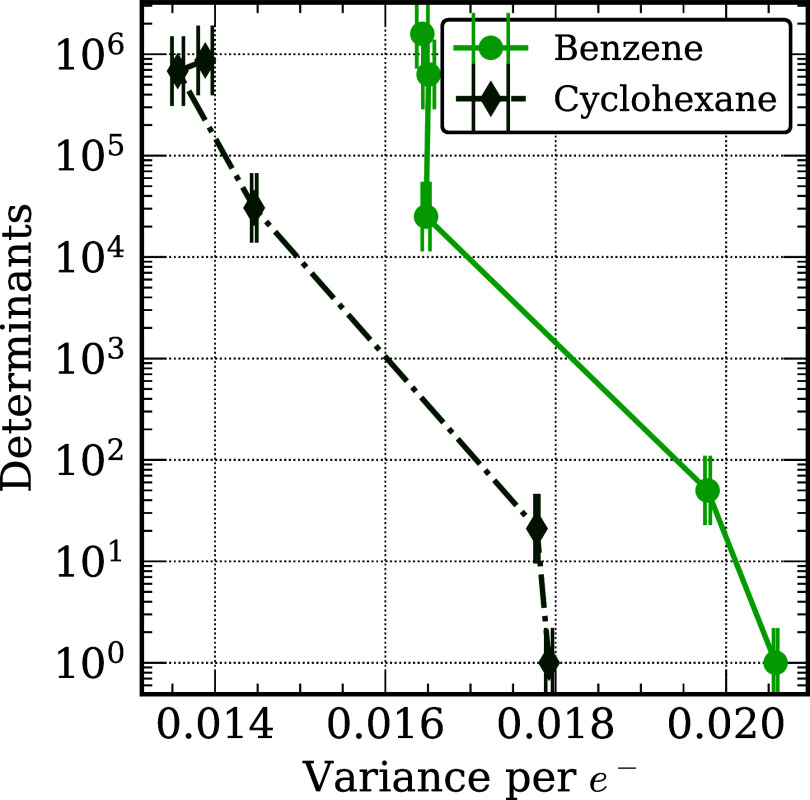
Variance per electron (Ha^2^) in the DMC energies of benzene and cyclohexane as a function of the number of Slater determinants in the trial wave functions.

The results of the calculations on benzene and cyclohexane suggest the following strategy for reducing the fixed-node bias in the DMC energies: namely employing SD trial wave functions for the hydrogenated species and multideterminant trial wave functions with a truncation of 0.001 on the coefficient of the multideterminant expansion for the dehydrogenated species. To test this strategy, six additional systems were treated with the results being shown in Figure 5. The figure also reports the corresponding results for benzene and cyclohexane. As seen from this figure, the errors in the DMC values of the hydrogenation energies of six of the seven systems considered are significantly reduced when employing a truncation of 0.001 on the CI coefficients for the unsaturated species. Moreover, for four of the systems, there is a small reduction in the error when employing a truncation of 0.01.

**Figure 5:**
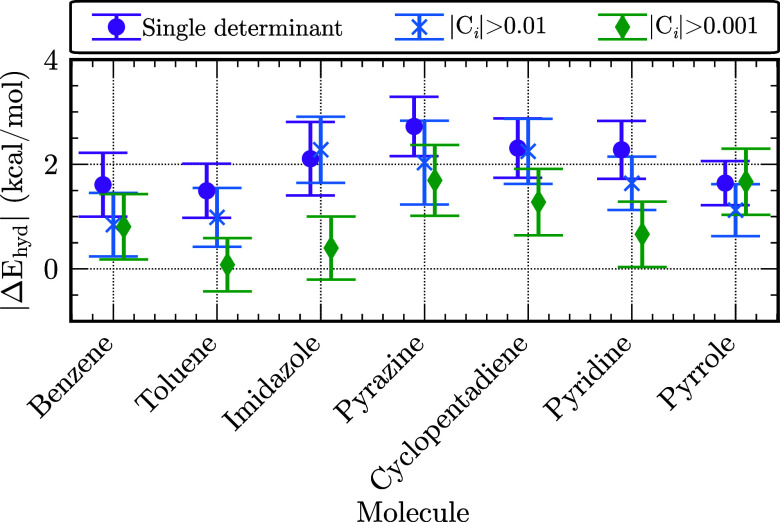
Differences (kcal/mol) between DMC and DLPNO-CCSD­(T) values of Δ*E*_hyd_ as a function of the number of determinants in the trial wave functions for the subset of seven systems.

#### Optimizing the Orbitals of the Trial Wave Functions for DMC

We next turn to orbital optimization as a way to improve the nodal surface of the SD trial wave function. For this test we use benzene and pyrazine together with their hydrogenated products. In generating the orbital optimized trial wave functions, the coefficients of the occupied orbitals were optimized together with the parameters in the Jastrow factors using the VMC procedure and allowing rotations among the 200 lowest lying (occupied and unoccupied) orbitals. The resulting trial wave functions were then employed in SD-DMC calculations on benzene pyrazine and their hydrogenated products. As seen from the results reported in Table 2 the errors in the hydrogenation energies from SD-DMC calculations are not reduced within statistical errors when using the trial wave functions with the VMC-optimized orbitals.

**Table 2:** Differences in the Values of Δ*E*_hyd_ (kcal/mol H_2_) from SD-DMC, with and without Orbital Optimization, from the DLPNO-CCSD­(T) Results

	benzene	pyrazine
SD-DMC	1.64 ± 0.53	2.40 ± 0.53
orbital optimized SD-DMC	1.76 ± 0.25	2.42 ± 0.24
MD-DMC	0.81 ± 0.62	1.96 ± 0.68

### Comparison with Experiment

In this section the calculated values of Δ*H*_hyd_ are compared with available experimental values listed in Table S1. For this comparison, the calculated reaction energies were corrected for ZPE, thermal, and enthalpic contributions. Figure 6 reports the deviations of the Δ*H*_hyd_ values from the ωB97M-V, SD-DMC, and DLPNO-CCSD­(T) calculations from experiment. A summary of the MAE of each of these methods, as well as B3LYP, over the entire test set is presented in Table 3. For both ωB97M-V and DLPNO-CCSD­(T) the MAEs of the calculated Δ*H*_hyd_ values with respect to experiment are less than 0.9 kcal/mol. The corresponding MAEs for B3LYP and SD-DMC calculations are 2.8 and 1.5 kcal/mol, respectively. For most of the systems considered the SD-DMC calculations underestimate the heat of reaction per H_2_ molecule by ∼2 kcal/mol, consistent with the errors caused by the fixed node bias discussed earlier in the manuscript. For 4-aminopyridine the SD-DMC, DLPNO-CCSD­(T) and the ωB97M-V all give a value of the heat of reaction considerably larger than the experimental value, suggesting an error in the experimental result in this case.

**Figure 6:**
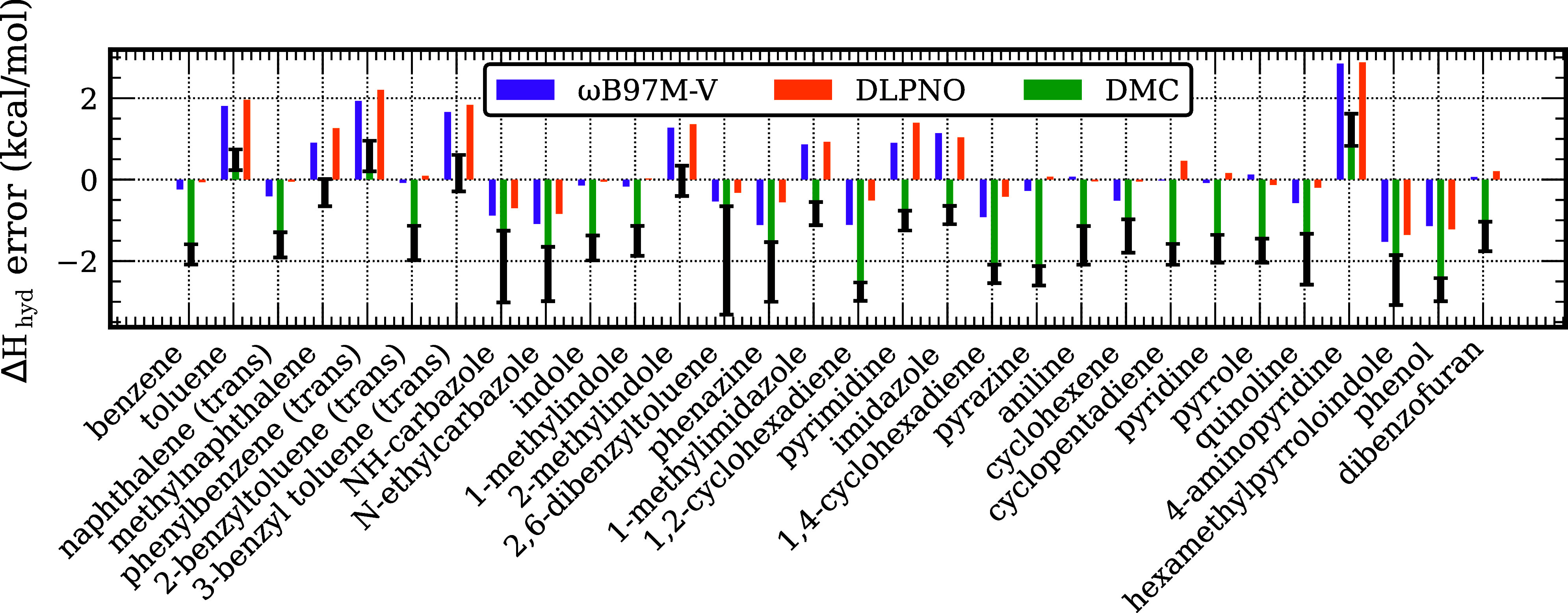
Differences between the calculated (DFT with the ωB97M-V functional, SD-DMC, and DLPNO-CCSDT) and experimental values of Δ*H*_hyd_ per mol H_2_. The “(trans)” label indicates that the trans isomer of the hydrogenated species was used.

**Table 3:** MAEs in the Calculated Heats of Reaction per mol H_2_ Relative to the Experimental Values^a^

method	MAE kcal/mol
ωB97M-V	0.82
B3LYP	2.85
SD-DMC	1.56
DLPNO-CCSD(T)	0.75

a The calculated results include corrections for vibrational ZPE and thermal corrections.

## Conclusions

In this work, the performance of various electronic structure methods for describing hydrogenation energies of LOHCs was explored. Specifically, the methods considered were DLPNO-CCSD­(T), DFT with the ωB97M-V and B3LYP functionals, and DMC using single determinant, multideterminant, and orbital optimized trial wave functions. We find that both DLPNO-CCSD­(T) and the ωB97M-V density functional methods give Δ*E*_hyd_ values in good agreement with experimental data, with MAEs less than 0.9 kcal/mol H_2_.

Single determinant DMC calculations result in a MAEs of 1.6 kcal/mol compared to the experiment. It is shown that using small multideterminant trial functions for the unsaturated molecules can reduce the MAE in the DMC Δ*E*_hyd_ values to under 1 kcal/mol H_2_. In contrast, optimizing the orbitals for the trial wave function in VMC did not make a significant impact on the resulting SD-DMC values of the hydrogenation energies.

Although DFT calculations with the ωB97M-V functional give hydrogenation energies in good agreement with the results of DLPNO-CCSD­(T) and with multideterminant DMC, it is not clear that it will perform as well for other LOHC candidates as it has for those examined here. For systems that resemble the candidate molecules included in this data set, DFT calculations employing the ωB97M-V functional are expected to provide a low-cost approach for screening LOHC candidates. For more challenging systems, DLPNO-CCSD­(T) or multideterminant DMC calculations may be required.

## Supplementary Material



## Data Availability

The data that support the findings of this study are openly available in the Materials Data Facility^106,107^ at 10.18126/p2h4-yb73.^108^

## Notes

Summary of the experimental enthalpies of hydrogenation, comparison between localized coupled cluster methods, analysis of the CIPSI selected configuration interaction results, and complete set of computed hydrogenation energies (PDF)

## #

High Energy Density Physics Theory, Sandia National Laboratories, Albuquerque, NM 87185, United States

## ∇

Department of Chemistry, University of Washington, Seattle, WA 98195, United States.

## ○

Qubit Pharmaceuticals Inc., Boston, MA 02116, United States.

## References

[1] Cabana J., Alaan T., Crabtree G. W., Huang P.-W., Jain A., Murphy M., N’Diaye J., Ojha K., Agbeworvi G., Bergstrom H., Gersib S., Harb H., Stejer A., Quiles-Galarza G., Rodriguez O., Caruso I., Gon ¸calves J. M., Chen G. Y., Fernández C. A., Pan H., Ritter K., Yang Y., Zhang H., García-Álvarez A. C., Ilic S., Kumar K., Silcox R., Yao Y., Song H., Stoyanov S., Saraf M., Chen C. H., Subasinghe S. M. S., Gomes R., Lang S., Murphy E., Thind A. S., Zheng Y. (2022). NGenE 2022: Electrochemistry for
decarbonization. ACS Energy Lett..

[2] Preuster P., Papp C., Wasserscheid P. (2017). Liquid organic
hydrogen carriers
(LOHCs): Toward a hydrogen-free hydrogen economy. Acc. Chem. Res..

[3] Crabtree G. W., Dresselhaus M. S., Buchanan M. V. (2004). The hydrogen economy. Phys. Today.

[4] Jones, L. Perspectives on the evolution into a hydrogen economy. Miami Univ. First World Hydrogen Energy Conf. Proc,. 1976.

[5] Bockris J. O. M. (2013). The
hydrogen economy: Its history. Int. J. Hydrogen
Energy.

[6] Sekine Y., Higo T. (2021). Recent trends on the dehydrogenation catalysis of liquid organic
hydrogen carrier (LOHC): A review. Top. Catal..

[7] Perreault P., Van Hoecke L., Pourfallah H., Kummamuru N. B., Boruntea C.-R., Preuster P. (2023). Critical challenges
towards the commercial
rollouts of a LOHC-based H_2_ economy. Curr. Opin. Green Sustainable Chem..

[8] D’Ambra F., Gébel G. (2023). Literature
review: State-of-the-art hydrogen storage
technologies and liquid organic hydrogen carrier (LOHC) development. Sci. Technol. Energy Transition.

[9] Modisha P. M., Ouma C. N., Garidzirai R., Wasserscheid P., Bessarabov D. (2019). The prospect of hydrogen storage using liquid organic
hydrogen carriers. Energy fuels.

[10] Wei D., Shi X., Qu R., Junge K., Junge H., Beller M. (2022). Toward a hydrogen
economy: Development of heterogeneous catalysts for chemical hydrogen
storage and release reactions. ACS Energy Lett..

[11] Narayanan T. M., He G., Gençer E., Shao-Horn Y., Mallapragada D. S. (2022). Role of liquid hydrogen carriers
in deeply decarbonized
energy systems. ACS Sustainable Chem. Eng..

[12] Teichmann D., Arlt W., Wasserscheid P., Freymann R. (2011). A future energy supply
based on liquid organic hydrogen carriers (LOHC). Energy Environ. Sci..

[13] Makepeace J. W., He T., Weidenthaler C., Jensen T. R., Chang F., Vegge T., Ngene P., Kojima Y., de Jongh P. E., Chen P. (2019). Reversible ammonia-based and liquid organic hydrogen carriers for
high-density hydrogen storage: Recent progress. Int. J. Hydrogen Energy.

[14] Niermann M., Timmerberg S., Drünert S., Kaltschmitt M. (2021). Liquid organic
hydrogen carriers and alternatives for international transport of
renewable hydrogen. Renew. Sust. Energy Rev..

[15] He T., Pei Q., Chen P. (2015). Liquid organic
hydrogen carriers. J. Energy Chem..

[16] Teichmann D., Stark K., Müller K., Zöttl G., Wasserscheid P., Arlt W. (2012). Energy storage in residential
and
commercial buildings via liquid organic hydrogen carriers (LOHC). Energy Environ. Sci..

[17] Teichmann D., Arlt W., Wasserscheid P. (2012). Liquid organic hydrogen carriers
as an efficient vector for the transport and storage of renewable
energy. Int. J. Hydrogen Energy.

[18] Clot E., Eisenstein O., Crabtree R. H. (2007). Computational structure–activity
relationships in H_2_ storage: How placement of N atoms affects
release temperatures in organic liquid storage materials. Chem. Commun. (Cambridge, U. K.).

[19] Aakko-Saksa P. T., Cook C., Kiviaho J., Repo T. (2018). Liquid organic hydrogen
carriers for transportation and storing of renewable energy–review
and discussion. J. Power Sources.

[20] Von Wild, J. ; Friedrich, T. ; Cooper, A. ; Toseland, B. ; Muraro, G. ; TeGrotenhuis, W. ; Wang, Y. ; Humble, P. ; Karim, A. Liquid organic hydrogen carriers (LOHC): An auspicious alternative to conventional hydrogen storage technologies. 18th World Hydrogen Energy Conference, Essen, Germany, 2010.

[21] Cooper, A. C. ; Campbell, K. M. ; Pez, G. P. An integrated hydrogen storage and delivery approach using organic liquid-phase carriers. Proc. 16th World Hydrogen Energy Conference, Lyon, France, 2006.

[22] DOE technical targets for onboard hydrogen storage for light-duty vehicles; US Department of Energy: Washington, D.C., USA, 2017.

[23] Amende M., Kaftan A., Bachmann P., Brehmer R., Preuster P., Koch M., Wasserscheid P., Libuda J. (2016). Regeneration of LOHC
dehydrogenation catalysts: In-situ IR spectroscopy on single crystals,
model catalysts, and real catalysts from UHV to near ambient pressure. Appl. Surf. Sci..

[24] Cho J.-Y., Kim H., Oh J.-E., Park B. Y. (2021). Recent
advances in homogeneous/heterogeneous
catalytic hydrogenation and dehydrogenation for potential liquid organic
hydrogen carrier (LOHC) systems. Catalysts.

[25] Kim T. W., Jeong H., Baik J. H., Suh Y.-W. (2022). State-of-the-art
catalysts for hydrogen storage in liquid organic hydrogen carriers. Chem. Lett..

[26] Markiewicz M., Zhang Y., Bösmann A., Brückner N., Thöming J., Wasserscheid P., Stolte S. (2015). Environmental and health
impact assessment of liquid organic hydrogen carrier (LOHC) systems–challenges
and preliminary results. Energy Environ. Sci..

[27] Markiewicz M., Zhang Y.-Q., Empl M. T., Lykaki M., Thöming J., Steinberg P., Stolte S. (2019). Hazard assessment of quinaldine-,
alkylcarbazole-, benzene-and toluene-based liquid organic hydrogen
carrier (LOHCs) systems. Energy Environ. Sci..

[28] Niermann M., Beckendorff A., Kaltschmitt M., Bonhoff K. (2019). Liquid Organic Hydrogen
Carrier (LOHC)–Assessment based on chemical and economic properties. Int. J. Hydrogen Energy.

[29] Rao P. C., Yoon M. (2020). Potential liquid-organic
hydrogen carrier (LOHC) systems: A review
on recent progress. Energies.

[30] Niermann M., Drünert S., Kaltschmitt M., Bonhoff K. (2019). Liquid organic hydrogen
carriers (LOHCs)–techno-economic analysis of LOHCs in a defined
process chain. Energy Environ. Sci..

[31] Kariya N., Fukuoka A., Ichikawa M. (2002). Efficient
evolution of hydrogen from
liquid cycloalkanes over Pt-containing catalysts supported on active
carbons under “wet–dry multiphase conditions”. Appl. Catal. A Gen.

[32] Okada Y., Sasaki E., Watanabe E., Hyodo S., Nishijima H. (2006). Development
of dehydrogenation catalyst for hydrogen generation in organic chemical
hydride method. Int. J. Hydrogen Energy.

[33] Crabtree R. H. (2017). Nitrogen-containing
liquid organic hydrogen carriers: Progress and prospects. ACS Sustainable Chem. Eng..

[34] Pez, G. P. ; Scott, A. R. ; Cooper, A. C. ; Cheng, H. (Air Products and Chemicals, Inc., Allentown, PA (United States)). Hydrogen storage by reversible hydrogenation of pi-conjugated substrates. US Patent. US 7101530 B2, 2006.

[35] Stark K., Keil P., Schug S., Müller K., Wasserscheid P., Arlt W. (2016). Melting points of potential liquid
organic hydrogen carrier systems consisting of N-alkylcarbazoles. J. Chem. Eng. Data.

[36] Huang B., Von Lilienfeld O. A. (2021). Ab initio machine learning in chemical
compound space. Chem. Rev..

[37] Montavon G., Rupp M., Gobre V., Vazquez-Mayagoitia A., Hansen K., Tkatchenko A., Müller K.-R., Von Lilienfeld O. A. (2013). Machine learning of molecular electronic
properties
in chemical compound space. New J. Phys..

[38] Ramakrishnan R., von Lilienfeld O. A. (2017). Machine
learning, quantum chemistry, and chemical space. Rev. Comput. Chem..

[39] Liu L., Zhu T., Li C., Zhao Y., Yang M., Ke H., Dong Y. (2023). Exploring the feasibility of 2,5-Dimethylindole as
a liquid organic
hydrogen carrier: A combined theoretical and experimental investigation. ACS Sustainable Chem. Eng..

[40] Izquierdo R., Cubillan N., Guerra M., Rosales M. (2021). Substituted
heterocycles
as new candidates for liquid organic hydrogen carriers: In silico
design from DFT calculations. Int. J. Hydrogen
Energy.

[41] Zhao Y., Truhlar D. G. (2011). Density Functional
Theory for Reaction Energies: Test
of Meta and Hybrid Meta Functionals, Range-Separated Functionals,
and Other High-Performance Functionals. J. Chem.
Theory Comput.

[42] Harb H., Elliott S. N., Ward L., Foster I. T., Klippenstein S. J., Curtiss L. A., Assary R. S. (2023). Uncovering
novel liquid organic hydrogen
carriers: A systematic exploration of chemical compound space using
cheminformatics and quantum chemical methods. Digital Discovery.

[43] Harb H., Elliott S. N., Ward L., Foster I. T., Klippenstein S. J., Curtiss L. A., Assary R. S. (2025). Accurate dehydrogenation enthalpies
dataset for liquid organic hydrogen carriers. Sci. Data.

[44] Khairbek, A. A. Quantum calculations to estimate the heat of hydrogenation theoretically. In Advanced Applications of Hydrogen and Engineering Systems in the Automotive Industry; Cocco, L. ; Aziz, M. , Eds.; IntechOpen, 2021.

[45] Foulkes W.
M. C., Mitas L., Needs R. J., Rajagopal G. (2001). Quantum Monte
Carlo simulations of solids. Rev. Mod. Phys..

[46] Dubecký M., Mitas L., Jurecka P. (2016). Noncovalent
interactions by quantum
Monte Carlo. Chem. Rev..

[47] Wagner L. K., Ceperley D. M. (2016). Discovering correlated
fermions using quantum Monte
Carlo. Rep. Prog. Phys..

[48] Tenti G., Nakano K., Tirelli A., Sorella S., Casula M. (2024). Principal
deuterium Hugoniot via quantum Monte Carlo and Δ-learning. Phys. Rev. B.

[49] Huang B., Von Lilienfeld O. A., Krogel J. T., Benali A. (2023). Toward DMC accuracy
across chemical space with scalable Δ-QML. J. Chem. Theory Comput..

[50] Dumi A., Upadhyay S., Bernasconi L., Shin H., Benali A., Jordan K. D. (2022). The binding of atomic hydrogen on graphene from density
functional theory and diffusion Monte Carlo calculations. J. Chem. Phys..

[51] Doblhoff-Dier K., Meyer J., Hoggan P. E., Kroes G.-J. (2017). Quantum Monte Carlo
Calculations on a Benchmark Molecule–Metal Surface Reaction:
H_2_ + Cu(111). J. Chem. Theory Comput..

[52] Fanta R., Bajdich M. (2025). Resolution of Selectivity
Steps of CO Reduction Reaction
on Copper by Quantum Monte Carlo. J. Phys. Chem.
Lett..

[53] Folmsbee D., Hutchison G. (2021). Assessing conformer energies using
electronic structure
and machine learning methods. Int. J. Quantum
Chem..

[54] Ruth M., Gerbig D., Schreiner P. R. (2023). Machine learning for bridging the
gap between density functional theory and coupled cluster energies. J. Chem. Theory Comput.

[55] Riplinger C., Sandhoefer B., Hansen A., Neese F. (2013). Natural triple
excitations
in local coupled cluster calculations with pair natural orbitals. J. Chem. Phys..

[56] Chai J.-D., Head-Gordon M. (2008). Long-range
corrected hybrid density functionals with
damped atom–atom dispersion corrections. Phys. Chem. Chem. Phys..

[57] Becke A. D. (1988). Density-functional
exchange-energy approximation with correct asymptotic behavior. Phys. Rev. A.

[58] Lee C., Yang W., Parr R. G. (1988). Development of the Colle-Salvetti
correlation-energy formula into a functional of the electron density. Phys. Rev. B.

[59] Vosko S. H., Wilk L., Nusair M. (1980). Accurate spin-dependent electron
liquid correlation energies for local spin density calculations: A
critical analysis. Can. J. Phys..

[60] Stephens P. J., Devlin F. J., Chabalowski C. F., Frisch M. J. (1994). Ab Initio calculation
of vibrational absorption and circular dichroism spectra using density
functional force fields. J. Phys. Chem..

[61] Zhao Y., Truhlar D. G. (2008). The M06 suite of
density functionals for main group
thermochemistry, thermochemical kinetics, noncovalent interactions,
excited states, and transition elements: two new functionals and systematic
testing of four M06-class functionals and 12 other functionals. Theor. Chem. Acc..

[62] Mardirossian N., Head-Gordon M. (2016). ωB97M-V: A combinatorially
optimized, range-separated
hybrid, meta-GGA density functional with VV10 nonlocal correlation. J. Chem. Phys..

[63] Veccham S. P., Head-Gordon M. (2021). Assessment
of Performance of Density Functionals for
Predicting Potential Energy Curves in Hydrogen Storage Applications. J. Phys. Chem. A.

[64] Santra G., Martin J. M. L. (2019). Some observations on the performance
of the most recent
exchange-correlation functionals for the large and chemically diverse
GMTKN55 benchmark. AIP Conf. Proc..

[65] Epstein A. R., Spotte-Smith E. W. C., Venetos M. C., Andriuc O., Persson K. A. (2023). Assessing
the Accuracy of Density Functional Approximations for Predicting Hydrolysis
Reaction Kinetics. J. Chem. Theory Comput..

[66] Weininger D., Weininger A., Weininger J. L. (1989). SMILES. 2. Algorithm for generation
of unique SMILES notation. J. Chem. Inf. Comput.
Sci..

[67] Weininger D. (1988). SMILES, a
chemical language and information system. 1. Introduction to methodology
and encoding rules. J. Chem. Inf. Comput. Sci..

[68] Landrum, G. ; Tosco, P. ; Kelley, B. ; Rodriguez, R. ; Cosgrove, D. ; Vianello, R. ; sriniker ,; Gedeck, P. ; Jones, G. ; NadineSchneider ,; Kawashima, E. ; Nealschneider, D. ; Dalke, A. ; Swain, M. ; Cole, B. ; Turk, S. ; Savelev, A. ; Hurst, T. ,; Vaucher, A. ; Wójcikowski, M. ; Take, I. ; Scalfani, V. F. ; Walker, R. ; Ujihara, K. ; Probst, D. ; Lehtivarjo, J. ; Faara, H. ; Godin, G. ,; Pahl, A. ; Monat, J. rdkit/rdkit: 2024_09_6 (Q3 2024) Release.

[69] Rappe A. K., Casewit C. J., Colwell K. S., Goddard W. A. I., Skiff W. M. (1992). UFF, a
full periodic table force field for molecular mechanics and molecular
dynamics simulations. J. Am. Chem. Soc..

[70] Grimme S., Bannwarth C., Shushkov P. (2017). A robust and accurate tight-binding
quantum chemical method for structures, vibrational frequencies, and
noncovalent interactions of large molecular systems parametrized for
all spd-block elements (Z = 1–86). J.
Chem. Theory Comput.

[71] Pracht P., Bohle F., Grimme S. (2020). Automated
exploration of the low-energy
chemical space with fast quantum chemical methods. Phys. Chem. Chem. Phys..

[72] Krishnan R., Binkley J. S., Seeger R., Pople J. A. (1980). Self-consistent
molecular orbital methods. XX. A basis set for correlated wave functions. J. Chem. Phys..

[73] Hehre W. J., Ditchfield R., Pople J. A. (1972). Self-consistent molecular orbital
methods. XII. Further extensions of gaussian-type basis sets for use
in molecular orbital studies of organic molecules. J. Chem. Phys..

[74] Frisch M. J., Pople J. A., Binkley J. S. (1984). Self-consistent molecular orbital
methods 25. Supplementary functions for Gaussian basis sets. J. Chem. Phys..

[75] Hay B., Hameury J., Davee G., Grelard M. (2014). Assessment of uncertainties
in calibration of langavant calorimeters. Int.
J. Thermophys..

[76] Fowler J., Alpert B., O’Neil G., Swetz D., Ullom J. (2022). Energy calibration
of nonlinear microcalorimeters with uncertainty estimates from gaussian
process regression. Journal of low temperature
physics.

[77] Skliarov, V. ; Muentean, K. ; Timofeev, E. Calibration of primary measuring converter by using of modeling and experimental evaluation of the initial data. 18th International Congress of Metrology, 2017; p 08005.

[78] Kilday M. V. (1980). Systematic
errors in an isoperibol solution calorimeter measured with standard
reference reactions. J. Res. Natl. Bur. Stand.
(U. S.).

[79] Dunning T. H. (1989). Gaussian basis
sets for use in correlated molecular
calculations. I. The atoms boron through neon and hydrogen. J. Chem. Phys..

[80] Woon D. E., Dunning T. H. (1993). Gaussian basis sets for use in correlated
molecular calculations. III. The atoms aluminum through argon. J. Chem. Phys..

[81] Pritchard B. P., Altarawy D., Didier B., Gibsom T. D., Windus T. L. (2019). A new basis
set exchange: An open, up-to-date resource for the molecular sciences
community. J. Chem. Inf. Model..

[82] Feller D. (1996). The role of
databases in support of computational chemistry calculations. J. Comput. Chem..

[83] Schuchardt K. L., Didier B. T., Elsethagen T., Sun L., Gurumoorthi V., Chase J., Li J., Windus T. L. (2007). Basis set
exchange:
A community database for computational sciences. J. Chem. Inf. Model..

[84] Sun Q. (2015). Libcint: An
efficient general integral library for gaussian basis functions. J. Comput. Chem..

[85] Sun Q., Berkelbach T. C., Blunt N. S., Booth G. H., Guo S., Li Z., Liu J., McClain J. D., Sayfutyarova E. R., Sharma S., Wouters S., Chan G. K.-L. (2018). PySCF: the Python-based
simulations of chemistry framework. WIREs Comput.
Mol. Sci..

[86] Sun Q., Zhang X., Banerjee S., Bao P., Barbry M., Blunt N. S., Bogdanov N. A., Booth G. H., Chen J., Cui Z.-H., Eriksen J. J., Gao Y., Guo S., Hermann J., Hermes M. R., Koh K., Koval P., Lehtola S., Li Z., Liu J., Mardirossian N., McClain J. D., Motta M., Mussard B., Pham H. Q., Pulkin A., Purwanto W., Robinson P. J., Ronca E., Sayfutyarova E. R., Scheurer M., Schurkus H. F., Smith J. E. T., Sun C., Sun S.-N., Upadhyay S., Wagner L. K., Wang X., White A., Whitfield J. D., Williamson M. J., Wouters S., Yang J., Yu J. M., Zhu T., Berkelbach T. C., Sharma S., Sokolov A. Y., Chan G. K.-L. (2020). Recent developments in the PySCF program package. J. Chem. Phys..

[87] Dunning T. H. (1989). Gaussian basis
sets for use in correlated molecular
calculations. I. The atoms boron through neon and hydrogen. J. Chem. Phys..

[88] Kendall R. A., Dunning T. H., Harrison R. J. (1992). Electron affinities
of the first-row atoms revisited. Systematic basis sets and wave functions. J. Chem. Phys..

[89] Neese F., Wennmohs F., Becker U., Riplinger C. (2020). The ORCA quantum
chemistry program package. J. Chem. Phys..

[90] Rolik Z., Kállay M. (2011). A general-order local coupled-cluster method based
on the cluster-in-molecule approach. J. Chem.
Phys..

[91] Szabó P. B., Csóka J., Kállay M., Nagy P. R. (2023). Linear-scaling local
natural orbital CCSD­(T) approach for open-shell systems: algorithms,
benchmarks, and large-scale applications. J.
Chem. Theory Comput.

[92] Sorathia K., Frantzov D., Tew D. P. (2024). Improved CPS and
CBS Extrapolation
of PNO-CCSD­(T) Energies: The MOBH35 and ISOL24 Data Sets. J. Chem. Theory Comput..

[93] Wappett D. A., Goerigk L. (2024). Exploring CPS-Extrapolated DLPNO–CCSD­(T1)
Reference
Values for Benchmarking DFT Methods on Enzymatically Catalyzed Reactions. J. Phys. Chem. A.

[94] Mester D., Nagy P. R., Csóka J., Gyevi-Nagy L., Szabó P. B., Horváth R. A., Petrov K., Hégely B., Ladóczki B., Samu G., Lőrincz B. D., Kállay M. (2025). Overview of
Developments in the MRCC Program System. J.
Phys. Chem. A.

[95] Kállay M., Nagy P. R., Mester D., Rolik Z., Samu G., Csontos J., Csóka J., Szabó P. B., Gyevi-Nagy L., Hégely B., Ladjánszki I., Szegedy L., Ladóczki B., Petrov K., Farkas M., Mezei P. D., Ganyecz (2020). The MRCC program system: Accurate
quantum chemistry
from water to proteins. J. Chem. Phys..

[96] Anderson J. B. (1980). Quantum
chemistry by random walk: Higher accuracy. J.
Chem. Phys..

[97] Jastrow R. (1955). Many-body
problem with strong forces. Phys. Rev..

[98] Huron B., Malrieu J. P., Rancurel P. (1973). Iterative
perturbation calculations
of ground and excited state energies from multiconfigurational zeroth-order
wavefunctions. J. Chem. Phys..

[99] Bennett M. C., Melton C. A., Annaberdiyev A., Wang G., Shulenburger L., Mitas L. (2017). A New Generation of
Effective Core Potentials for Correlated Calculations. J. Chem. Phys..

[100] Quantum Monte Carlo Methods . https://qmcpack.readthedocs.io/en/develop/methods.html#diffusion-monte-carlo, accessed: May 19, 2025.

[101] Umrigar C.
J., Toulouse J., Filippi C., Sorella S., Hennig R. G. (2007). Alleviation of the
Fermion-sign problem by optimization
of many-body wave functions. Phys. Rev. Lett..

[102] Kim J., Baczewski A. D., Beaudet T. D., Benali A., Bennett M. C., Berrill M. A., Blunt N. S., Borda E. J. L., Casula M., Ceperley D. M., Chiesa S., Clark B. K., Clay R. C., Delaney K. T., Dewing M., Esler K. P., Hao H., Heinonen O., Kent P. R. C., Krogel J. T., Kylänpää I., Li Y. W., Lopez M. G., Luo Y., Malone F. D., Martin R. M., Mathuriya A., McMinis J., Melton C. A., Mitas L., Morales M. A., Neuscamman E., Parker W. D., Flores S. D. P., Romero N. A., Rubenstein B. M., Shea J. A. R., Shin H., Shulenburger L., Tillack A. F., Townsend J. P., Tubman N. M., Goetz B. V. D., Vincent J. E., Yang D. C., Yang Y., Zhang S., Zhao L. (2018). QMCPACK: An open source ab initio quantum Monte Carlo package for
the electronic structure of atoms, molecules and solids. J. Phys.: Condens. Matter.

[103] Kent P. R. C., Annaberdiyev A., Benali A., Bennett M. C., Landinez Borda E. J., Doak P., Hao H., Jordan K. D., Krogel J. T., Kylänpää I., Lee J., Luo Y., Malone F. D., Melton C. A., Mitas L., Morales M. A., Neuscamman E., Reboredo F. A., Rubenstein B., Saritas K., Upadhyay S., Wang G., Zhang S., Zhao L. (2020). QMCPACK: Advances in the development, efficiency, and application
of auxiliary field and real-space variational and diffusion quantum
Monte Carlo. J. Chem. Phys..

[104] Garniron Y., Applencourt T., Gasperich K., Benali A., Ferté A., Paquier J., Pradines B., Assaraf R., Reinhardt P., Toulouse J., Barbaresco P., Renon N., David G., Malrieu J. P., Véril M., Caffarel M., Loos P. F., Giner E., Scemama A. (2019). Quantum Package
2.0: An open-source determinant-driven suite of programs. J. Chem. Theory Comput..

[105] Dash M., Feldt J., Moroni S., Scemama A., Filippi C. (2019). Excited states with selected configuration interaction-quantum
Monte Carlo: Chemically accurate excitation energies and geometries. J. Chem. Theory Comput..

[106] Blaiszik B., Ward L., Schwarting M., Gaff J., Chard R., Pike D., Chard K., Foster I. (2019). A Data Ecosystem to Support Machine Learning in Materials
Science. MRS Commun..

[107] Blaiszik B., Chard K., Pruyne J., Ananthakrishnan R., Tuecke S., Foster I. (2016). The Materials Data Facility: Data
Services to Advance Materials Science Research. JOM.

[108] Dumi, A. ; Upadhyay, S. ; Harb, H. ; Assary, R. S. ; Sorescu, D. C. ; Jordan, K. D. ; Benali, A. Dataset for Electronic Structure Methods for Predicting the Hydrogenation Energies of Candidate Molecules for Hydrogen Storage, 2025, 10.18126/p2h4-yb73 PMC12670397 41234149

