# Supplementary material for: A Comparison of Electronic Structure Methods for Predicting the Hydrogenation Energies of Candidate Molecules for Hydrogen Storage
Source: J Phys Chem A. 2025 Nov 14;129(47):11003–13. doi: 10.1021/acs.jpca.5c05284 (PMC12670397; doi:10.1021/acs.jpca.5c05284)
Supplement: Supplementary file 1 [file jp5c05284_si_001.pdf]

Supporting Information for: "Electronic Structure Methods for Predicting the Hydrogenation Energies of Candidate Molecules for Hydrogen Storage"

Amanda Dumi,[†,‡] Shiv Upadhyay,[¶,‡] Hassan Harb,[§] Rajeev S. Assary,[§] Dan C. Sorescu,[∥,⊥] Kenneth D. Jordan,[‡,⊥] and Anouar Benali[*,#,@]

†*Current: High Energy Density Physics Theory, Sandia National Laboratories, Albuquerque, NM 87185*

‡*Department of Chemistry, University of Pittsburgh, Pittsburgh, Pennsylvania 15260, United States*

¶*Current: Department of Chemistry, University of Washington, Seattle, WA 98195*

§*Material Science Division, Argonne National Laboratory, Argonne, IL 60439, United States*

∥*National Energy Technology Laboratory, United States Department of Energy, Pittsburgh, Pennsylvania 15236, United States*

⊥*Department of Chemical and Petroleum Engineering, University of Pittsburgh, Pittsburgh, Pennsylvania 15261, United States*

#*Current: Qubit Pharmaceuticals Inc, Boston, MA 02116, USA*

@*Computational Science Division, Argonne National Laboratory, Argonne, IL 60439, United States*

E-mail: abenali.sci@gmail.com

Summary of molecular details and experimental ΔH_{hyd} values from the literature.

Table S1: Structures given as SMILES strings and experimental enthalpies of hydrogenation (kcal/mol) per mol of H_2. The "(trans)" label indicates that the trans isomer of the hydrogenated species was used.

Index	Dehydrogenated Molecule Name	Dehydrogenated Species	Hydrogenated Species	ΔH_{hyd} (kcal/mol)
1	benzene	C1=CC=CC=C1	C1CCCCC1	−16.40[1]
2	toluene	CC1=CC=CC=C1	CC1CCCCC1	−16.32[1]
3	naphthalene (trans)	C1=CC=C2C=CC=CC2=C1	C1CCC2CCCCC2C1	−15.94[1]
4	methylnaphthalene	CC1=CC2=CC=CC=C2C=C1	CC1CC2CCCCC2CC1	−15.61[2]
5	phenylbenzene (trans)	C1=CC=C(C=C1)C2=CC=CC=C2	C1CCC(CC1)C2CCCCC2	−15.92[3]
6	2-benzyltoluene (trans)	CC1=CC=CC=C1CC2=CC=CC=C2	CC1CCCCC1CC2CCCCC2	−15.18[2]
7	3-benzyl toluene (trans)	CC1=CC(=CC=C1)CC2=CC=CC=C2	CC1CC(CCC1)CC2CCCCC2	−16.97[2]
8	NH-carbazole	C1=CC=C2C(=C1)C3=CC=CC=C3N2	C1CCC2C(C1)C3CCCCC3N2	−12.21[4]
9	N-ethylcarbazole	C1=CC=C2C(=C1)C3=CC=CC=C3N2(CC)	C1CCC2C(C1)C3CCCCC3N2(CC)	−12.09[4]
10	indole	C1=CC=C2C(=C1)C=CN2	C1CCC2C(C1)CCN2	−13.52[5]
11	1-methylindole	CN1C=CC2=CC=CC=C21	CN1CCC2CCCCC21	−12.40[6]
12	2-methylindole	CC1=CC2=CC=CC=C2N1	CC1CC2CCCCC2N1	−13.19[5]
13	2,6-dibenzyltoluene	CC1=C(C=CC=C1CC2=CC=CC=C2)CC3=CC=CC=C3	CC1C(CCCC1CC2CCCCC2)CC3CCCCC3	−15.63[2]
14	phenazine	C1=CC=C2C(=C1)N=C3C=CC=CC3=N2	C1CCC2C(C1)NC3CCCCC3N2	−14.65[7]
15	1-methylimidazole	CN1C=CN=C1	CN1CCNC1	−9.35[3]
16	1,2-cyclohexadiene	C1CC=CC=C1	C1CCCCC1	−27.20[8]
17	pyrimidine	C1=CN=CN=C1	C1CNCNC1	−14.36[3]
18	Imidazole	N1C=CN=C1	N1CCNC1	−9.51[3]
19	1,4-cyclohexadiene	C1C=CCC=C1	C1CCCCC1	−27.20[8]
20	pyrazine	C1=CN=CC=N1	C1CNCCN1	−13.60[9]
21	aniline	C1=CC=C(C=C1)N	C1CCC(CC1)N	−15.30[8]
22	cyclohexene	C1CCC=CC1	C1CCCCC1	−28.40[8]
23	cyclopentadiene	C1C=CC=C1	C1CCCC1	−25.70[3]
24	pyridine	C1=CC=NC=C1	C1CCNCC1	−14.90[3]
25	pyrrole	C1=CNC=C1	C1CNCC1	−13.40[3]
26	quinoline	C1=CC=C2C(=C1)C=CC=N2	C1CCC2C(C1)CCCN2	−14.79[2]
27	4-aminopyridine	C1=CN=CC=C1N	C1CNCCC1N	−13.36[3]
28	1,2,3,5,6,7-hexamethylpyrrolo[3,2-f]indole	C1=C3C(=CC2=C1[N](C(=C2C)C)C)C(=C([N]3C)C)C	C1C3C(CC2C1[N](C(C2C)C)C)C(C([N]3C)C)C	−9.73[3]
29	phenol	C1=CC=C(C=C1)O	C1CCC(CC1)O	−14.58[3]
30	dibenzofuran	C1=CC=CC2=C1OC3=C2C=CC=C3	C1CCCC2C1OC3C2CCCC3	−13.55[3]

Agreement between LNO-CC and DLPNO-CC methods for benzene.

Table S2: ΔE_{hyd} per mole of H_2 for benzene for the approximate coupled cluster (CC) methods explored.

Method	ΔE_{hyd} (kcal/mol)
DLPNO-CCSD	-25.80
DLPNO-CCSD(T)	-24.72
LNO-CCSD	-25.89
LNO-CCSD(T)	-24.45
LNO-CCSDT	-24.60

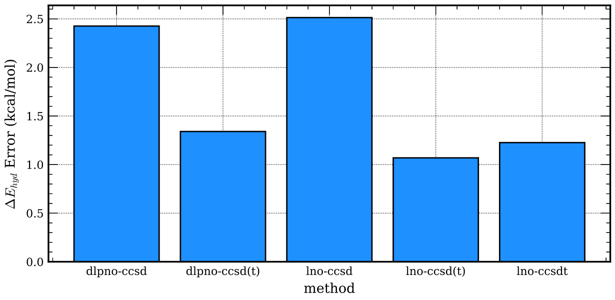

Figure S1: Error in ΔE_{hyd} per mole of H_2 against experimental values calculated with various CC methods. zero-point energy (ZPE) and thermal corrections from B3LYP are included.

CIPSI determinant expansion details

Table S3: Number of determinants kept at different truncation values on the determinant coefficients for the dehydrogenated species.

Molecule	Truncation 0.01	Truncation 0.001
benzene	21	24.568
toluene	42	24,237
13-pyrazole	84	22,254
14-diazene	123	23,031
cyclopentadiene	39	22,942
pyridine	62	25,462
pyrrole	89	24,230

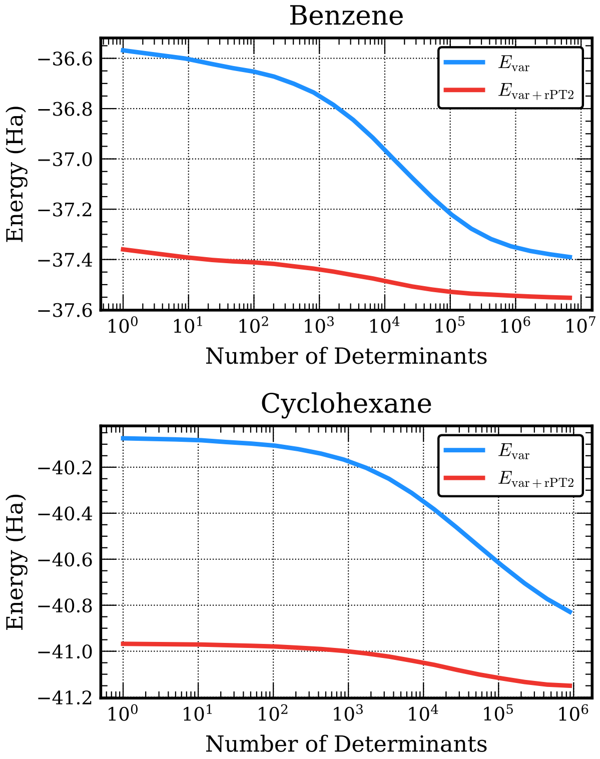

Figure S2: A comparison of the CIPSI energies of benzene and cyclohexane as a function of the number of Slater determinants in the variational space. The dehydrogenated species (benzene) requires a larger determinant expansion than the hydrogenated species (cyclohexane) to reach convergence.

Summary of ΔE_{hyd} values across methods

Table S4: ΔE_{hyd} values for the various methods without ZPE or thermal corrections in kcal/mol H_2. The numerical index of each molecule is defined in Table S1

Index	DLPNO-CCSD(T)	B3LYP	ωB97M-V	DMC
1	−23.52	−21.06	−23.69	−25.28±0.25
2	−21.44	−18.77	−21.59	−22.91±0.26
3	−23.04	−20.26	−23.39	−24.59±0.31
4	−21.42	−18.53	−21.77	−23.00±0.33
5	−20.81	−18.13	−21.08	−22.43±0.38
6	−22.24	−19.56	−22.41	−23.89±0.42
7	−22.38	−19.71	−22.56	−24.06±0.45
8	−20.03	−16.97	−20.21	−21.46±0.88
9	−19.95	−16.91	−20.20	−21.42±0.67
10	−20.68	−17.77	−20.77	−22.31±0.31
11	−19.43	−16.53	−19.63	−20.96±0.37
12	−18.96	−15.94	−19.05	−20.35±0.37
13	−23.10	−20.46	−23.31	−24.76±1.33
14	−22.39	−19.49	−22.95	−24.10±0.73
15	−15.63	−13.60	−15.69	−17.39±0.28
16	−35.02	−33.33	−35.62	−37.26±0.23
17	−20.28	−18.46	−20.77	−22.68±0.24
18	−15.94	−13.78	−15.84	−17.85±0.23
19	−34.95	−33.42	−35.45	−36.84±0.23
20	−20.82	−18.68	−21.17	−23.25±0.24
21	−22.45	−19.41	−22.34	−24.02±0.47
22	−35.70	−34.18	−36.17	−37.04±0.41
23	−32.82	−31.35	−33.30	−35.11±0.26
24	−21.89	−19.58	−22.13	−23.74±0.34
25	−20.81	−18.43	−20.55	−22.42±0.30
26	−22.11	−19.39	−22.48	−23.86±0.63
27	−17.75	−14.85	−17.78	−19.40±0.39
28	−18.26	−14.89	−18.43	−19.37±0.61
29	−22.91	−20.06	−22.83	−24.40±0.28
30	−20.48	−17.57	−20.61	−22.07±0.36

Table S5: Total ZPE and thermal corrections values in kcal/mol calculated at the ωB97M-V/aug-cc-pVQZ level of theory. The (d) and (h) denote dehydrogenated and hydrogenated species, respectively.

Index	ZPE (d)	thermal (d)	ZPE (h)	thermal (h)
1	63.16	2.77	106.88	3.59
2	80.43	3.91	124.32	4.62
3	92.72	4.31	165.63	5.58
4	109.99	5.42	183.16	6.56
5	114.33	5.58	201.95	7.28
6	149.41	6.88	237.02	8.96
7	149.08	6.53	236.82	9.04
8	111.24	5.63	199.35	6.97
9	146.70	7.44	234.22	8.75
10	81.77	3.94	140.29	5.04
11	99.21	4.98	157.63	5.95
12	99.01	5.05	157.73	6.03
13	217.96	9.06	349.67	11.75
14	107.15	5.76	210.20	7.53
15	62.43	3.36	91.97	3.84
16	77.04	3.24	106.89	3.59
17	48.61	2.62	93.14	3.39
18	45.01	2.33	74.87	2.98
19	76.95	3.30	106.92	3.58
20	48.47	2.60	92.88	3.46
21	73.72	3.64	117.65	4.41
22	91.96	3.46	106.87	3.59
23	58.28	2.60	88.36	3.26
24	55.95	2.67	99.91	3.52
25	52.13	2.45	81.56	3.16
26	85.49	4.18	158.70	5.49
27	66.46	3.54	110.68	4.49
28	204.24	11.23	278.70	11.61
29	65.92	3.46	109.80	4.31
30	103.63	5.34	191.38	7.10

DLPNO-CCSD(T) complete-PNO-space extrapolation

The complete PNO-space extrapolation was done using the CPS(6,7) approach.[10,11] The difference relative to the TightPNO settings is minimal.

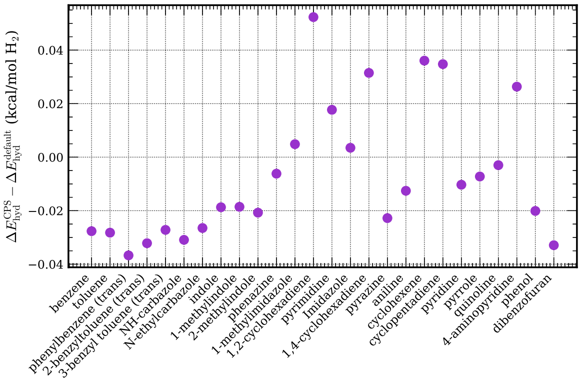

Figure S3: Difference between the DLPNO-CCSD(T) hydrogenation energies as compared to the complete PNO-space extrapolation from those calculated with the default TightPNO TCutPNO parameter of 1e-7[12]

Approximating anharmonic effects using scaled vibrational frequencies

The recommended scaling factors for the ωB97M-V functional were used to estimate anharmonic effects.[13] These results indicate that the anharmonic effects are minimal in this system, and the results in the main text use the unscaled frequencies.

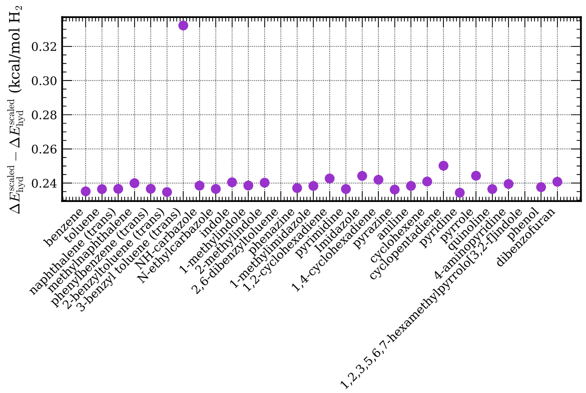

Figure S4: Difference between the DLPNO-CCSD(T) hydrogenation energies calculated with scaled and unscaled frequencies.

Basis set dependence of the DFT Hydrogenation energies

The basis set dependence of the hydrogenation energies computed with the ωB97M-V functional The complete basis set limit was taken using a simple two point extrapolation using the aug-cc-pVTZ and aug-cc-pVQZ basis sets. The resulting hydrogenation energies were compared to the ωB97M-V/cc-pVQZ results in the paper, and these were found to be extremely similar.

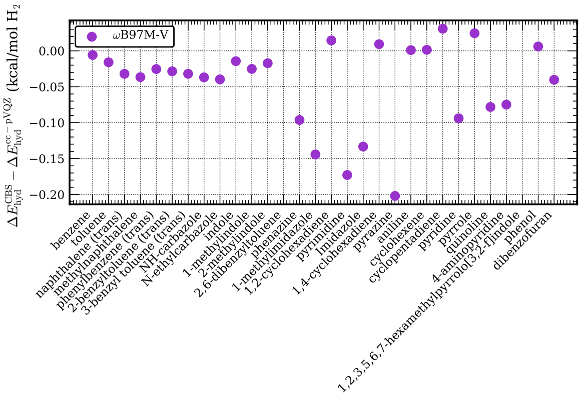

Figure S5: Difference between the ωB97M-V hydrogenation energies at the CBS limit relative to the cc-pVQZ basis set results.

Impact of molecular geometry of the DLPNO Hydrogenation energies

For a subset of the molecules DLPNO-CCSD(T) calculations were carried out using the ωB97M-V. These calculations gave hydrogenation energies very close to those obtained using the B3LYP optimized geometries.

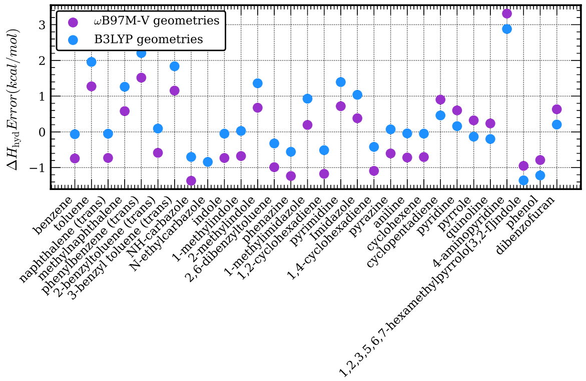

Figure S6: The impact of molecular geometries used in the DLPNO-CCSD(T) calculations as quantified through a comparison with the experimental hydrogenation enthalpies.

References

(1) Kariya, N.; Fukuoka, A.; Ichikawa, M. Efficient evolution of hydrogen from liquid cycloalkanes over Pt-containing catalysts supported on active carbons under "wet–dry multiphase conditions". *Appl. Catal. A Gen* **2002**, *233*, 91–102.

(2) Aakko-Saksa, P. T.; Cook, C.; Kiviaho, J.; Repo, T. Liquid organic hydrogen carriers for transportation and storing of renewable energy–review and discussion. *J. Power Sources* **2018**, *396*, 803–823.

(3) He, T.; Pei, Q.; Chen, P. Liquid organic hydrogen carriers. *J. Energy Chem.* **2015**, *24*, 587–594.

(4) Biniwale, R. B.; Rayalu, S.; Devotta, S.; Ichikawa, M. Chemical hydrides: a solution to high capacity hydrogen storage and supply. *Int. J. Hydrogen Energy* **2008**, *33*, 360–365.

(5) Konnova, M. E.; Li, S.; Bösmann, A.; Müller, K.; Wasserscheid, P.; Andreeva, I. V.; Turovtzev, V.; Zaitsau, D. H.; Pimerzin, A. A.; Verevkin, S. P. Thermochemical Properties and Dehydrogenation Thermodynamics of Indole Derivates. *Ind. Eng. Chem. Res.* **2020**, *59*, 20539–20550.

(6) Rao, P. C.; Yoon, M. Potential liquid-organic hydrogen carrier (LOHC) systems: A review on recent progress. *Energies* **2020**, *13*, 6040.

(7) Niermann, M.; Beckendorff, A.; Kaltschmitt, M.; Bonhoff, K. Liquid Organic Hydrogen Carrier (LOHC)–Assessment based on chemical and economic properties. *Int. J. Hydrogen Energy* **2019**, *44*, 6631–6654.

(8) Linstorm, P. NIST chemistry webbook, NIST standard reference database number 69. *J. Phys. Chem. Ref. Data, Monograph* **1998**, *9*, 1–1951.

(9) Clot, E.; Eisenstein, O.; Crabtree, R. H. Computational structure–activity relationships in H_2 storage: How placement of N atoms affects release temperatures in organic liquid storage materials. *Chem. Commun. (Cambridge, U. K.)* **2007**, 2231–2233.

(10) Sorathia, K.; Frantzov, D.; Tew, D. P. Improved CPS and CBS Extrapolation of PNO-CCSD(T) Energies: The MOBH35 and ISOL24 Data Sets. *J. Chem. Theory Comput.* **2024**, *20*, 2740–2750.

(11) Wappett, D. A.; Goerigk, L. Exploring CPS-Extrapolated DLPNO–CCSD(T1) Reference Values for Benchmarking DFT Methods on Enzymatically Catalyzed Reactions. *J. Phys. Chem. A* **2024**, *128*, 62–72.

(12) Altun, A.; Neese, F.; Bistoni, G. Extrapolation to the Limit of a Complete Pair Natural Orbital Space in Local Coupled-Cluster Calculations. *Journal of Chemical Theory and Computation* **2020**, *16*, 6142–6149.

(13) Liang, J.; Feng, X.; Liu, X.; Head-Gordon, M. Analytical harmonic vibrational frequencies with VV10-containing density functionals: Theory, efficient implementation, and benchmark assessments. *The Journal of Chemical Physics* **2023**, *158*, 204109.
